# Body composition changes in male patients with chronic obstructive pulmonary disease: Aging or disease process?

**DOI:** 10.1371/journal.pone.0180928

**Published:** 2017-07-10

**Authors:** Li-Wen Lee, Chieh-Mo Lin, Hung-Chou Li, Pei-Lin Hsiao, An-Chi Chung, Chu-Jung Hsieh, Pi-Chi Wu, Shu-Feng Hsu

**Affiliations:** 1 Department of Diagnostic Radiology, Chang Gung Memorial Hospital, Chiayi, Taiwan; 2 Department of Nursing, Chang Gung University of Science and Technology, Chiayi, Taiwan; 3 Division of Pulmonary and Critical Care medicine, Chang Gung Memorial Hospital, Chiayi, Taiwan; 4 Graduate Institute of Clinical Medical Sciences, College of Medicine, Chang Gung University, Taoyuan, Taiwan; 5 Department of Respiratory Therapy, Chang Gung Memorial Hospital, Chiayi, Taiwan; 6 Department of Nursing, Chang Gung Memorial Hospital, Chiayi, Taiwan; National and Kapodistrian University of Athens, GREECE

## Abstract

**Background:**

Chronic obstructive pulmonary disease (COPD) mainly affects middle-age and elderly adults. It is unclear if the presence of muscle wasting and fat accumulation in patients with COPD is age or disease-related. This study investigated the effect of age and COPD disease severity on body composition with the aim of identifying a biomarker(s) for COPD.

**Methods:**

Healthy subjects and patients with COPD of different severity were recruited. Dual-energy X-ray absorptiometry was used to analyze total and segmental body composition. Subjects included in the analysis were classified into four groups: healthy young (aged 20–45 years) (n = 35), healthy old (aged ≥ 60 years) (n = 37), moderate COPD (n = 40), and severe COPD (n = 14).

**Results:**

In healthy old adults, leg and limb lean masses were lower by 10.6% and 8.5%, respectively, compared with healthy young adults (P < 0.05). Appendicular lean outcomes were significantly lower in the moderate COPD compared to the healthy old group and were significant lower in subjects with severe COPD compared to those with moderate COPD. All fat depots were similar for both young and old healthy subjects and subjects with moderate COPD, but significantly decreased in patients with severe COPD.

**Conclusions:**

This study examined the changes in total and segmental body composition with aging and COPD severity. It found that aging and COPD altered the body composition differently, and the effect was most pronounced in leg lean mass. Remarkably, differences in appendicular lean masses were seen in mild COPD although no changes in body weight or BMI were apparent compared with healthy young adults. In contrast, fat depot changes were only observed in severe COPD. Aging and COPD processes are multifactorial and additional longitudinal studies are required to explore both the quantitative and qualitative changes in body composition with aging and disease process.

## Introduction

Chronic obstructive pulmonary disease (COPD) is a group of diseases characterized by progressive airflow limitation, airway inflammation and extrapulmonary effects [1]. Evidence of extrapulmonary systemic effects includes muscle wasting, osteoporosis, increased risk of cardiovascular disease, and increased inflammatory markers in the circulation [2, 3]. The prevalence of skeletal muscle wasting and dysfunction is 15% to 40% in patients with COPD, depending on the disease severity, leading to physical disability, exercise intolerance, and deaths [4, 5].

Traditionally, body weight and body mass index (BMI) are used to determine cachexia in COPD. However, this approach may underestimate the muscle wasting, as increased fat mass may obscure the loss of muscle mass in patients with less severe COPD [6, 7]. In patients with COPD, the mechanical efficiency and exercise capacity of the upper and lower limbs are differentially affected, with the upper limbs being less affected than the lower limbs [8], highlighting the need to monitor both total and segmental body composition changes in patients with COPD.

The aging process is associated with an increased in body fat predominantly in the abdominal region and a decline in lean mass, with and without change in BMI [9]. A progressive decline in skeletal muscle mass and strength is defined as sarcopenia. According to the European Working Group on Sarcopenia in Older People [10], when aging is the only contributing factor to sarcopenia in older population, it is called primary sarcopenia or age-related sarcopenia and when sarcopenia is secondary to an underlying disease such as COPD, malnutrition, cancer or organ failure, it is classified as secondary sarcopenia. COPD mainly affects middle-aged and older adults, therefore, the skeletal muscle changes in individuals with COPD may due to the aging process and disease progression of COPD.

Cross sectional modalities such as computerized tomography (CT) and magnetic resonance imaging (MRI) can be used to measure the volume of visceral and subcutaneous fat tissues. However, evaluation of the entire body using CT and MRI is laborious and time consuming. An alternative method is single sliced fat segmentation at the L4-5 disc level or umbilical level. However, tailored equations may be needed to assess specific age, gender, ethnicity and disease outcomes. Furthermore, the use of CT involves ionizing radiation and is not recommended for healthy subjects.

Dual energy X-ray absorptiometry (DXA) is a standard tool for measuring body composition in clinical practice. It can measure body composition parameters from total and segmental body levels using only background ionizing radiation [11–13]. In addition, DXA can provide lean, fat, and bone mass in regional and whole body levels with high accuracy, and therefore, is recommended for patients with COPD [14].

Both age and COPD are related to changes in body composition and therefore, it may be difficult to specifically investigate whether the observed body composition changes are age-related or disease-related. This study recruited healthy individuals across different ages as controls and compared their body composition to patients with different stages of COPD using DXA. We aimed to investigate differences in body composition parameters associated with aging and COPD severity to identify effective biomarker(s) for COPD.

## Materials and methods

### Study design

This cross-sectional study was approved by the Institutional Review Board of the Chang Gung Memorial Hospital (103-0635A3 and 103-1516B), and all patients gave their written informed consent. Subjects were recruited between July 2014 and September 2016. The study consisted of one clinic visit. Participants were asked to fast for at least two hours before reporting to the Chang Gung Memorial Hospital (Chiayi branch). Vigorous activities and alcohol were avoided for ≥ 48 hours before the study day. On arrival, participants were asked to void and change into a hospital gown. Body weight and height were measured to the nearest 0.1 kg and 0.1 cm, respectively, using a digital scale (Super-View, HW-3050, Taipei, Taiwan). The total study time was about one hour.

### Study subjects

Healthy young volunteers (aged 20 to 45 years), healthy old volunteers (aged ≥ 60 years) and COPD patients were recruited. Only male subjects were recruited. Healthy volunteers were recruited via hospital advertisements and word of mouth. Subjects with previously diagnosed COPD were recruited from the outpatient clinics and inpatient wards of our respiratory medicine department. Exclusion criteria included female individuals, the inability to lie flat or still, existing metal implants, cancer, and neurodegenerative disease.

### Pulmonary function test

In healthy young adults (aged 20 to 45 years), lung function testing was not performed but none of the subjects reported symptoms of cardiopulmonary disorders, such as shortness of breath, chronic cough, wheezing, and chest tightness. In healthy old adults (≥ 60 years of age), pre-bronchodilator lung function test was performed using a hand-held spirometer (MasterScreen Pneumo, CareFusion, Hochberg, Germany). The instrument was calibrated daily at the start of the experiment. Healthy old subjects with a pre-bronchodilator forced expiratory volume in one second/ forced vital capacity (FEV_1_/FVC) < 70% were regarded as having obstructive lung disease and were excluded from the study.

In subjects with established COPD, the diagnosis and classification of COPD was based on post-bronchodilator FEV_1_ and FVC using a commercial spirometer (Spirobank II, Medical International Research, Roma, Italy), according to the 2017 Global Initiative for Chronic Obstructive Lung Disease Guideline [15]. The classification was as followed: GOLD 1 (mild), FEV_1_/FVC < 70% and FEV_1_ ≥ 80% predicted; GOLD 2 (moderate), FEV_1_/FVC < 70% and 50% ≤ FEV_1_ < 80% predicted; GOLD 3 (severe), FEV_1_/FVC < 70% and 30% ≤ FEV_1_ <50% predicted; and GOLD 4 (very severe), FEV_1_/FVC < 70% and FEV_1_ < 30%.

### Dual-energy X-ray absorptiometry (DXA)

Body composition was assessed using a fan-beam DXA scanner equipped with software version 12.5 (Delphi A, Hologic, Bedford, MA, USA). Daily quality control of the device with a Hologic step phantom was performed before scanning a subject. The in vivo precision of the measurement of body composition at the whole-body level was 1.0%, according to product specifications. Whole body and segmental body composition, including lean, fat, and bone mass was analyzed. For each DXA scan, subjects were supine on the scanning bed with arms pronated by their side, legs were internally rotated by about 25°, and toes touched and fixed using strapping tape [16]. All DXA scans were analyzed by a single operator who followed the manufacturer’s instructions to define the regional boundaries on the two-dimensional DXA images and to divide the whole body into the head, trunk, right arm, left arm, right leg, and left leg segments [17]. Limb mass was calculated as the sum of masses in both arms and legs. The skeletal muscle index (SMI) was calculated using the following equation:

$$\text{SMI }=\text{limb lean mass in kg }÷{\left(\text{height in meter}\right)}^{2}$$

Percentage change of body composition from baseline was calculated as follows:

Percentage change =(absolute change value÷baseline value)×100%,

where baseline value was defined as that of young healthy group

### Statistical analysis

The statistical software PRISM 6 (GraphPad Software, Inc., San Diego, CA, USA) was used for one-way analysis of variance (ANOVA) and graph generation. Statistical differences between groups were tested using ANOVA with Bonferroni’s multiple comparisons test. Stepwise regression analysis was applied to identify predictors for predicting body composition variables using IBM SPSS Statistics version 22.0 (IBM Corp., NY, USA). Candidate predictors included age and disease severity (0: no COPD, 1: GOLD 2, 2: GOLD 3). Predict variables were included in the regression equation only if significant. Statistical significance was defined at α = 0.05. Statistical significance was a P value of < 0.05.

## Results

The study recruited 135 male subjects: 35 young healthy adults (aged 20–45 years), 45 old healthy adults (aged ≥ 60 years), 40 moderate COPD patients (GOLD 2), 14 severe COPD patients (GOLD 3), and one very severe COPD (GOLD 4) patients. Of the recruited healthy old subjects, eight (17.8%) were excluded from the study due to abnormal lung function tests (FEV_1_/FVC < 70%). The one subject with GOLD 4 was also excluded as a single subject was not sufficient for meaningful evaluation. The final analysis included 126 male subjects in four groups: the healthy young (n = 35), healthy old (n = 37), GOLD 2 (n = 40) and GOLD 3 (n = 14) groups.

The subject characteristics are shown in Table 1. All demographics and body composition measurements significantly differed across groups except for body height (P = 0.2370, Table 1). To investigate the effect of age on body composition, the total and segmental body composition estimates in the healthy young and old adult groups were compared (Table 1**)**. The effect of COPD severity on total and segmental body composition was also investigated by comparing different parameters between healthy older adults and subjects with GOLD 2 or GOLD 3 (Table 2).

**Table 1:** Patient characteristics.

Group	Healthy young	Healthy old	GOLD 2	GOLD 3	ANOVA
**Subjects**	35	37	40	14	
**FEV**_**1**_**/FVC**	NA	81.01	56.10****	41.39****	<0.0001
**(%)**		(7.149)	(8.152)	(6.146)	
**Age (yrs)**	35.32	70.86****	74.03****	68.54****	<0.0001
	(6.209)	(5.096)	(8.539)	(8.816)	
**Height (cm)**	165.8	164.6	163.4	164.1	0.2370
	(2.660)	(5.455)	(6.249)	(5.342)	
**Weight (kg)**	69.05	68.12	66.29	56.41***	0.0006
	(9.429)	(9.247)	(10.83)	(8.558)	
**BMI**	25.09	25.15	24.89	20.89***	0.0011
	(3.276)	(3.250)	(4.262)	(2.489)	
**Lean**					
**Total**	52.31	50.53	46.69***	41.31****	<0.0001
	(5.599)	(5.457)	(6.274)	(5.780)	
**Trunk**	24.81	24.70	24.45	21.43**	0.0042
	(2.998)	(3.012)	(3.266)	(2.810)	
**Limb**	23.91	21.88*	18.74****	16.42****	<0.0001
	(2.629)	(2.326)	(3.039)	(3.180)	
**Leg**	17.69	15.81***	13.49****	11.71****	<0.0001
	(1.927)	(1.784)	(2.206)	(2.326)	
**Arm**	6.215	6.062	5.244****	4.708****	<0.0001
	(0.8507)	(0.9446)	(0.9115)	(0.8800)	
**SMI**	8.696	8.072*	7.020****	6.106****	<0.0001
	(0.9111)	(0.7242)	(1.091)	(1.172)	
**Fat**					
**Total**	15.92	16.79	16.84	11.76*	0.0106
	(5.351)	(5.244)	(4.858)	(4.652)	
**Trunk**	8.151	8.896	8.978	5.622	0.0122
	(3.559)	(3.715)	(3.231)	(2.570)	
**Limb**	6.709	6.794	6.812	5.123*	0.0241
	(1.936)	(1.759)	(1.830)	(2.097)	
**Leg**	4.899	4.701	4.769	3.606**	0.0165
	(1.368)	(1.258)	(1.258)	(1.386)	
**Arm**	1.810	2.091	2.041	1.516	0.0226
	(0.6698)	(0.6397)	(0.6460)	(0.7530)	
**Body fat (%)**	21.99	23.72	25.26*	20.89	0.0084
	(5.036)	(4.740)	(4.398)	(6.601)	
**Trunk fat (%)**	23.21	25.18	25.86	19.74	0.0122
	(6.544)	(6.546)	(5.709)	(7.079)	
**Bone**					
**Total bone**	2.472	2.408	2.220*	2.025***	0.0002
	(0.2717)	(0.3062)	(0.4197)	(0.4318)	
**BMD**	1.191	1.151	1.121*	1.079**	0.0052
	(0.08102)	(0.09497)	(0.1386)	(0.1146)	

Data were expressed in the form of mean (standard deviation).

Abbreviations: ANOVA, analysis of variance; BMD, bone mass density, BMI, body mass index; FEV_1_/FVC, forced expiratory volume in one second/forced vital capacity; GOLD, The Global Initiative for Chronic Obstructive Lung Disease; SMI, skeletal muscle mass index.

*, P < 0.05;

**, P < 0.01;

***, P < 0.001;

****, P < 0.0001 by one-way ANOVA with Bonferroni’s multiple comparisons test.

**Table 2:** Differences between older subjects with and without chronic obstructive pulmonary disease.

Group	Healthy old	GOLD 2	GOLD 3	ANOVA
**Subjects**	37	40	14	
**Height (cm)**	164.6	163.4	164.1	0.6401
	(5.455)	(6.249)	(5.342)	
**Weight (kg)**	68.12	66.29	56.41***	0.0011
	(9.247)	(10.83)	(8.558)	
**BMI**	25.15	24.89	20.89**	0.0009
	(3.250)	(4.262)	(2.489)	
**Lean**				
**Total**	50.53	46.69****	41.31****	<0.0001
	(5.457)	(6.274)	(5.780)	
**Trunk**	24.70	24.45	21.43**	0.0031
	(3.012)	(3.266)	(2.810)	
**Limb**	21.88	18.74****	16.42****	<0.0001
	(2.326)	(3.039)	(3.180)	
**Leg**	15.81	13.49****	11.71****	<0.0001
	(1.784)	(2.206)	(2.326)	
**Arm**	6.062	5.244***	4.708****	<0.0001
	(0.9446)	(0.9115)	(0.8800)	
**SMI**	8.072	7.020****	6.106****	<0.0001
	(0.7242)	(1.091)	(1.172)	
**Fat**				
**Total**	16.79	16.84	11.76**	0.0033
	(5.244)	(4.858)	(4.652)	
**Trunk**	8.896	8.978	5.622**	0.0043
	(3.715)	(3.231)	(2.570)	
**Limb**	6.794	6.812	5.123*	0.0094
	(1.759)	(1.830)	(2.097)	
**Leg**	4.701	4.769	3.606*	0.0119
	(1.258)	(1.258)	(1.386)	
**Arm**	2.091	2.041	1.516*	0.0189
	(0.6397)	(0.6460)	(0.7530)	
**Body fat (%)**	23.72	25.26	20.89	0.0184
	(4.740)	(4.398)	(6.601)	
**Trunk fat (%)**	25.18	25.86	19.74*	0.0076
	(6.546)	(5.709)	(7.079)	
**Bone**				
**Total bone**	2.408	2.220	2.025*	0.0047
	(0.3062)	(0.4197)	(0.4318)	
**BMD**	1.151	1.121	1.079	0.1487
	(0.09497)	(0.1386)	(0.1146)	

Data were expressed in the form of mean (standard deviation).

*, P < 0.05;

**, P < 0.01;

***, P < 0.001;

****, P < 0.0001 by one-way ANOVA with Bonferroni’s multiple comparisons test.

### Total body composition

The body weight, BMI, total lean mass, total fat mass and total bone mass were not statistically difference between healthy young and healthy old groups (Table 1), but statistically difference among healthy old, GOLD 2 and GOLD 3 groups was observed (Table 2).

### Segmental lean mass

**Fig 1:**
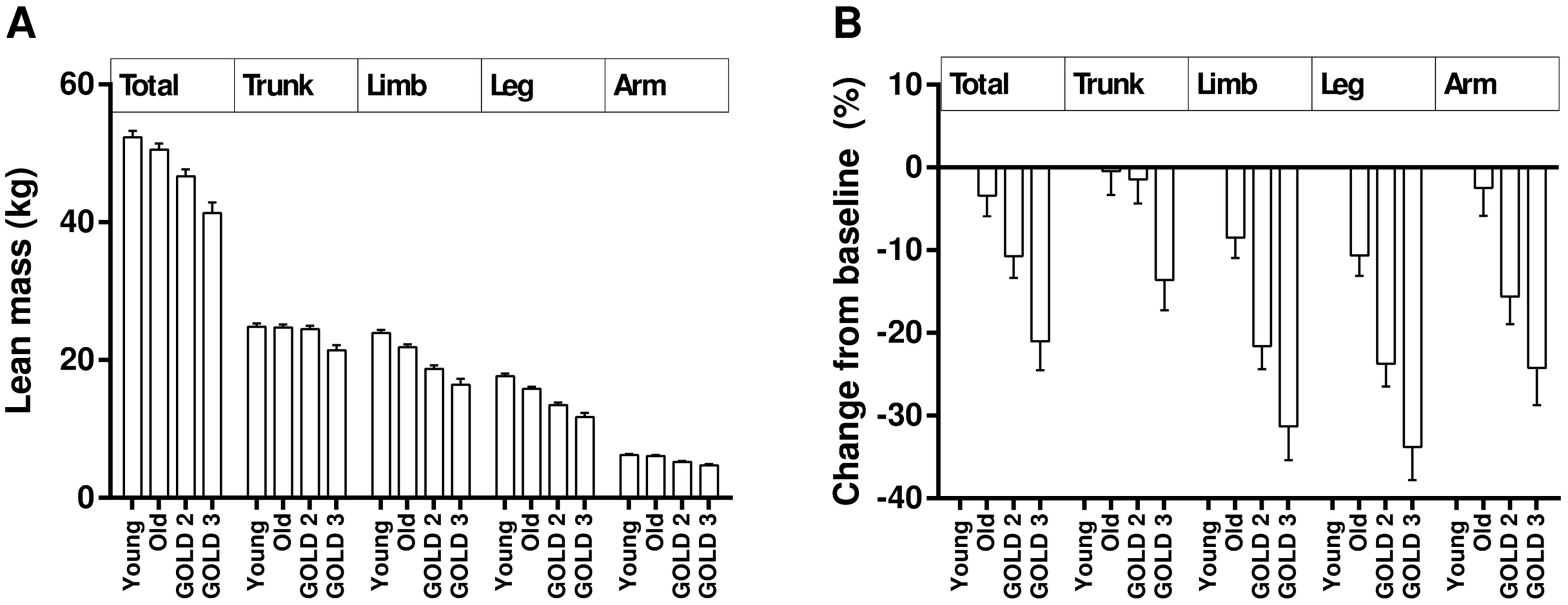
Changes in lean mass variables. (A) Total and segmental lean mass changes among groups. (B) Percentage changes of total and segmental lean mass variables compared with the healthy young adult group. Data were expressed as mean ± standard error of mean. Young, healthy young subjects aged 20–45 years; Old, healthy old subjects aged ≥ 60 years; GOLD, The Global Initiative for Chronic Obstructive Lung Disease.

In general, changes in the lean mass variables showed a similar trend with decreasing values with increasing age and COPD severity. The only exception was trunk lean mass (Fig 1). A one-way ANOVA with post-hoc test showed leg lean mass, limb lean mass, and SMI were significantly lower in healthy old adults compared to healthy young adults (P < 0.05, Table 1). In healthy old adults, leg and limb lean masses were lower by 10.6% and 8.5%, respectively, compared with healthy young adults. In contrast, arm lean mass was not significantly different between the two age groups. In this study, leg lean mass constituted approximately 73% of the total limb mass in healthy subjects. Therefore, differences in the limb lean and SMI between the two age groups mainly resulted from differences in leg lean rather than arm lean mass.

In older men, all lean-related parameters were significant different among subjects without COPD and with different stage of COPD (P ≤ 0.0031, Table 2). Post hoc analysis was performed to further explore the impact of disease severity on lean variables between the three groups. The limb-related lean outcomes were significantly lower in the GOLD 2 group compared with the healthy old group (P < 0.001, Table 2) and in subjects with GOLD 3 compared with those with GOLD 2 (P < 0.0001, Table 2). The trunk lean mass in subjects with GOLD 2 did not differ from those in healthy old subjects but was significantly higher than those with GOLD 3 (P < 0.01, Table 2). The results suggest limb-related lean mass become lower as COPD severity increases whereas trunk lean mass decreases only in severe COPD subjects.

### Fat depots

**Fig 2:**
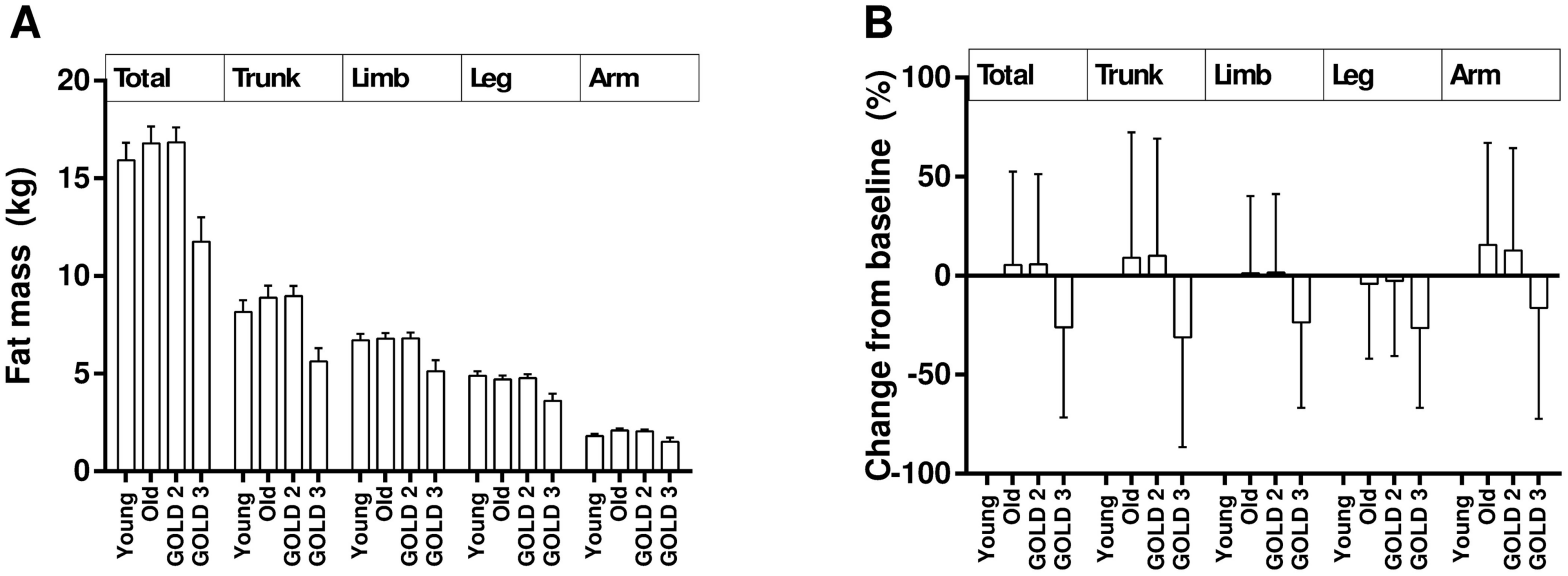
Changes in fat depots. (A) Total and segmental fat depot changes among groups. (B) Percentage changes of total and segmental fat mass variables from baseline of the healthy young adult group. Data were expressed in the form of mean ± standard error of mean.

For all fat depots, there was an overall significant difference between the four groups (P values ≤ 0.0241, Table 1). However, the changes exhibited different patterns for different fat depots (Fig 2). No age differences were found in all fat-related variables between younger and older healthy subjects (Table 1). However, all fat variables significantly differed between older healthy subjects and those with COPD (P values ≤ 0.0189, Table 2). Post hoc analysis found that subjects with GOLD 2 had a non-significant increase in all fat deposit parameters compared with older healthy adults, except for arm fat depot. However, in subjects with GOLD 3, significant reductions in all fat deposit parameters were observed compared with older health subjects and subjects with GOLD 2, suggesting that a reduction in all fat deposits is associated with more severe COPD. Similar findings were seen in the changes in percentage trunk fat and percentage total body fat (Table 2).

### Bone mass and bone mineral density

No age differences were found in the total bone mass and bone mineral density between younger and older healthy subjects (Table 1). In older subjects, a decrease in the bone mineral density with the progression of COPD was seen, but this did not reach statistical significance (P = 0.1487, Table 2). Subjects with GOLD 2 had a decrease in total bone mass compared with older healthy adults, but this did not reach statistical significance. However, in subjects with GOLD 3, significant reduction in total bone mass was observed compared with healthy old subjects, suggesting that a reduction in bone mass is associated with more severe COPD.

### Overall findings

**Fig 3:**
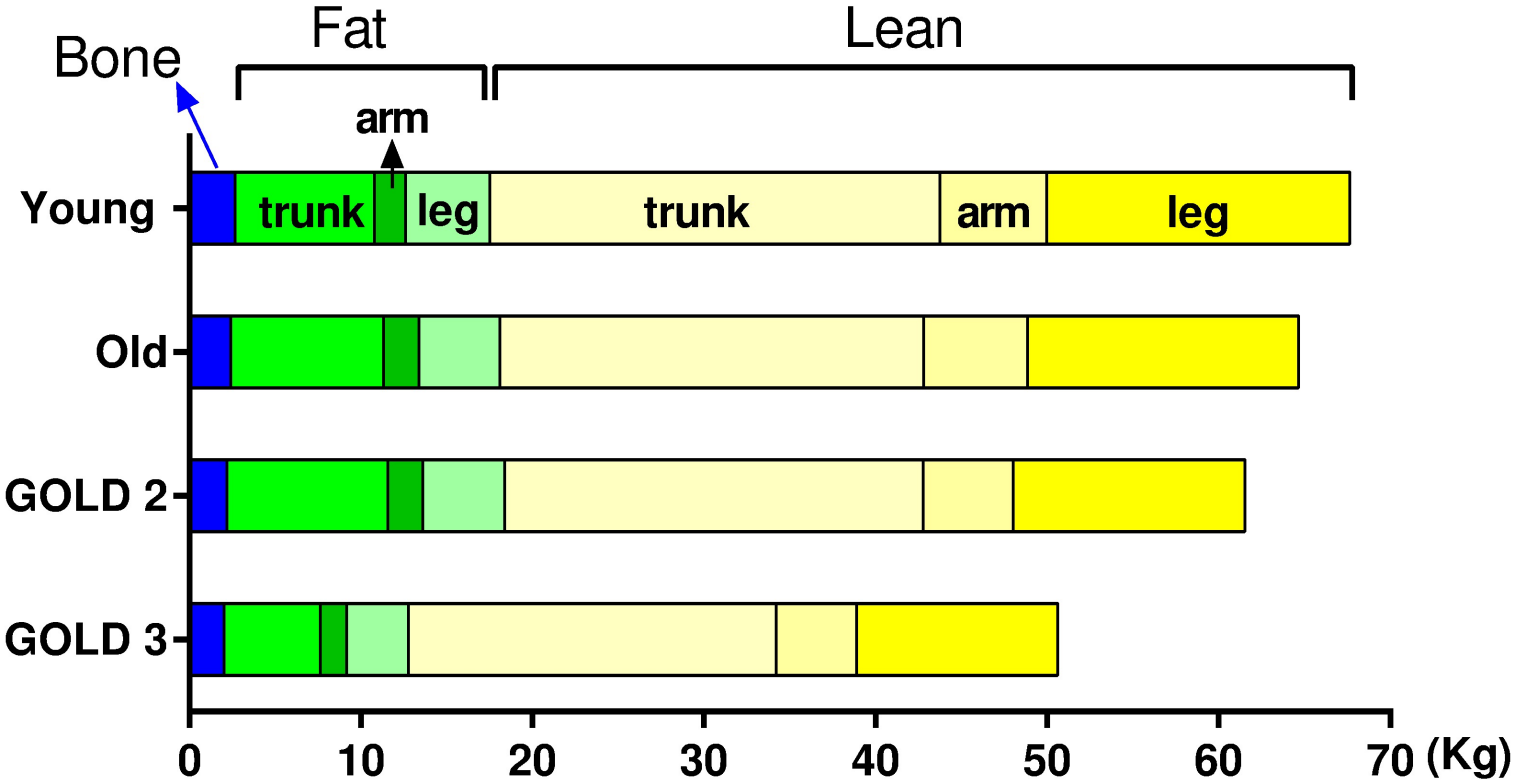
Summary of the dynamic changes in body composition among groups.

The dynamic changes in body composition in the four groups are summarized in Fig 3. The predominant change with increasing age was a decrease in leg lean mass whereas severe COPD was associated with a decrease in all the body composition variables. A summary of stepwise regression analysis is shown in Table 3 to identify predictors for body composition variables. Changes in the leg lean mass, limb lean mass and SMI could be explained by both age and COPD severity with an R^2^ of 0.515, 0.482 and 0.462, respectively. Furthermore, COPD severity could act a predictor for all lean-associated variables, total bone, BMD, total fat, limb fat and leg fat. Among the variables mentioned above, a moderate correlation (0.7 ≥ R > 0.5) between COPD severity and lean-associated estimators was observed, except for trunk lean mass (R = 0.267), and the correlations were weak (0.5 ≥ R > 0.3) between COPD severity and the other variables. Notably, neither age nor COPD severity were a predictor for the trunk fat, arm fat, percentage body fat and percentage trunk fat.

**Table 3:** Model summary of stepwise regression analysis.

					Change Statistics				
Model	R	R^2^	Adjusted R^2^	SEE	R^2^ Change	F Change	df1	df2	P value
**Lean**									
**Total**	.507^a^	.257	.251	5.79658	.257	42.993	1	124	.000
**Trunk**	.267^a^	.071	.064	3.09914	.071	9.480	1	124	.003
**Limb**	.647^a^	.418	.414	2.85823	.418	89.223	1	124	.000
	.694^b^	.482	.474	2.70801	.064	15.139	1	123	.000
**Leg**	.660^a^	.435	.430	2.15747	.435	95.470	1	124	.000
	.718^b^	.515	.507	2.00629	.080	20.391	1	123	.000
**Arm**	.509^a^	.259	.253	.89939	.259	43.446	1	124	.000
**SMI**	.646^a^	.418	.413	.98166	.418	89.000	1	124	.000
	.680^b^	.462	.453	.94752	.044	10.098	1	123	.002
**Fat**									
**Total**	.188^a^	.036	.028	5.19459	.036	4.566	1	124	.035
**Trunk**	-	-	-	-	-	-	-	-	-
**Limb**	.195^a^	.038	.030	1.89086	.038	4.898	1	124	.029
**Leg**	.215^a^	.046	.039	1.31664	.046	6.020	1	124	.016
**Arm**	-	-	-	-	-	-	-	-	-
**Body fat (%)**	-	-	-	-	-	-	-	-	-
**Trunk fat (%)**	-	-	-	-	-	-	-	-	-
**Bone**									
**Bone**	.384^a^	.147	.141	.35073	.147	21.436	1	124	.000
**BMD**	.284^a^	.081	.073	.10988	.081	10.857	1	124	.001

^a^Predictors: COPD severity (0: normal; 1: GOLD 2; 2: COLD 3)

^b^Predictors: age and COPD severity (0: normal; 1: GOLD 2; 2: COLD 3)

df: degree of freedom; SEE: standard error of estimate

## Discussion

Chronic obstructive pulmonary disease is more commonly found in middle age and older people and is associated with muscle wasting. Age can also impact muscle mass and body composition; hence it is not clear what changes in body composition is associated with COPD or is due to age. The aim of this study was to evaluate the differences in body composition between younger and older healthy subjects and older subjects with and without COPD. In healthy subjects, increasing age was only associated with a decrease in leg lean mass. No difference was observed with respect to arm and trunk lean mass or fat depots. In subjects with GOLD 2, both arm and leg lean mass decreased compared with healthy older subjects and no change was observed in the other variables. Cachexia appears more prevalent in advanced COPD [4, 7], and as expected, subjects with GOLD 3 had significant reduction in all body composition parameters compared with healthy subjects and subjects with GOLD 2, except for percentage body fat and BMD. Our study suggested that age and COPD severity primarily impacted lean variables rather than fat and bone variables.

It is well known that aging is associated with fat gain and lean mass loss. However, in this study, whole body composition estimates, including body weight, BMI, total body fat, total lean mass, total bone mass, and percent body fat, were similar between young and older healthy subjects. This may be due to selection bias, as the older adults who participated in this present study may have been more-functional and healthier than that of the general population [18]. Furthermore, there may be generational differences in body composition across age, with a positive trend in body fat and a negative trend in lean mass indices [19, 20]. Thus, a trend in generational differences may also affect the age-related changes in body composition, hence our findings might not reflect actual changes with aging [21]. Unfortunately, there have been no large-scaled, population-based studies using DXA to evaluate longitudinal changes in body composition across a wide range of ages. Additional studies are necessary to further investigate age-related changes in body composition.

Although no age-related difference was observed in body composition at whole body level, a significant difference in lean mass estimates was observed between younger and older healthy subjects at the level of body segments. Compared with younger subjects, leg and limb lean mass was significantly lower in older subjects. No difference was observed between groups for arm mass. Since leg lean mass constituted about 73% of the limb lean mass, the differences in limb lean mass between different age groups resulted mainly from differences in leg lean mass, suggesting loss of leg lean mass may be indicative of aging. This is in agreement with previous study by Janssen et al. which showed that aging was associated with a preferential decrease in skeletal muscle mass in the lower extremities [22].

In contrast to lean mass changes, all fat deposit variables were similarbetween the two different age groups. Our result are in agreement with the Florey Adelaide Male Aging Study which showed aging was related to a decrease in lean mass and a relative increase in regional percent of body fat [23]. However, the findings are inconsistent with other studies which found aging was related to fat accumulation and redistribution [24, 25]. The reasons why fat changes did not occur in the current study may be due to the small sample size in each group and/or to the selection bias of subjects, as we recruited healthy older individuals who may not represent the general population. Another possible explanation is the fat redistribution was mainly from subcutaneous to visceral and ectopic deposits during the aging process [26]. Since DXA is based on two-dimensional projection images, which cannot distinguish subcutaneous fat from visceral and ectopic fat, we may have missed changes in subcutaneous and visceral fat deposits.

This study identified two major types of changes in body composition associated with increasing COPD. First, all the limb lean-related estimates decreased with increasing COPD severity. Interestingly, trunk lean mass did not change with age and the development of GOLD 2 disease but did decrease with the development of GOLD 3 COPD. Of the lean mass variables assessed, trunk lean mass was the least affected by either aging or COPD severity. Second, all the fat deposits, were similar between older healthy adults and patients with GOLD 2 COPD. However, all fat deposit variables decreased significantly in subjects with GOLD 3 disease compared with healthy subjects and subjects with GOLD 2 COPD, with the one exception of the percentage of total body fat.

The role of fat changes as an indicator for disease severity in COPD remains controversial. Furuate R. et al. showed using CT that COPD patients have an increase in visceral fat as compared with controls [27]. However, their study did not compare body composition parameters with healthy subjects, as the control group included subjects with respiratory symptoms but with normal pulmonary function.

In this study, eight out of the 45 subjects (17.8%) were excluded from the healthy old subject group due to abnormal lung function tests with FEV_1_/FVC < 70%. According to a population-based prevalence study which reported that the prevalence of GOLD 1 was 5.9–16.3% in men [28], our healthy subjects seemed to have a higher prevalence of abnormal lung function. However, the healthy old subjects in the current study were only included with a pre-bronchodilator spirometer and a pre-bronchodilator FEV_1_/FVC < 70%, which may not meet the criteria for the diagnosis of COPD.

A range of factors, including age, race, sex, and some disease states can influence body composition in human. This study had the strength of recruiting patients with homogeneous race, gender, and height for investigating the effect of age and disease on the changes of body composition.

It should be acknowledged that the current study has some limitations. First, lung function test was not performed in the healthy young group. Studies showed that the prevalence of COPD was low in young adults in general population (3.2% to 6.4%) [29, 30], and the prevalence was significantly higher in those with respiratory symptoms. In the present study, none of the recruited young adults presented with respiratory symptoms and was at lower risk of COPD. Second, we only analyzed subjects with GOLD 2 and GOLD 3 COPD. We were not able to evaluate subjects with GOLD 1 or GOLD 4 disease due to the lack of subjects with these disease severities. Mild COPD subjects are rare in clinical settings because they experience only a slight reduction in air flow, and therefore, they may not realize that their lung function is impaired. On the other hand, subjects with GOLD 4 usually experience severe shortness of breath and have difficulty lying flat during DXA scan. In addition, we were only able to screen the healthy old subjects with pre-bronchodilator pulmonary function, and it is possible that some included healthy subjects may have had pulmonary restriction. Third, this study also did not divided subjects with COPD into emphysematous and bronchitis subtypes owing to the small sample size. The study did not take into account the cohort effect and the study was not longitudinal in design. Four, this study only examined the quantity of fat, lean, and bone masses but not their quality and/or function of these tissues. This may have impacted the findings, since both age and COPD can alter the power and strength of the lean mass [9, 31, 32].

## Conclusion

This study examined changes in total and segmental body composition with aging and COPD severity. The results indicate that aging and COPD altered body composition differently,and the effect was most pronounced in leg lean mass. Remarkably, differences in appendicular lean masses were seen in GOLD 2 patients, although no changes in body weight of BMI were apparent compared with healthy young adults. In contrast, fat depot changes were only observed in GOLD 3 patients. Aging and COPD processes are multifactorial and complex and therefore, additional longitudinal studies are required to explore both the quantitative and qualitative changes in body composition with aging and disease process.
